# *Notes from the Field:* Ketamine Detection and Involvement in Drug Overdose Deaths — United States, July 2019–June 2023

**DOI:** 10.15585/mmwr.mm7344a4

**Published:** 2024-11-07

**Authors:** Alana M. Vivolo-Kantor, Christine L. Mattson, Maria Zlotorzynska

**Affiliations:** 1 Division of Overdose Prevention, National Center for Injury Prevention and Control, CDC.

**Summary**

**What is already known about this topic?**

Expanded availability of ketamine for management of treatment-resistant depression has resulted in increased use.

**What is added by this report?**

During July 2019–June 2023, ketamine was detected in <1% of overdose deaths and was the only drug involved in 24 deaths. During this period, the percentage of overdose deaths with ketamine detected in toxicology reports increased from 0.3% (47 deaths) to 0.5% (107 deaths). Approximately 82% of deaths with ketamine detected in toxicology reports involved other substances, including illegally manufactured fentanyls, methamphetamine, or cocaine.

**What are the implications for public health practice?**

Further investigation is needed to better understand the role of ketamine in drug overdoses, particularly when multiple substances are used before death.

Ketamine, a Schedule III controlled substance* that is Food and Drug Administration (FDA)–approved for general anesthesia, can produce mild hallucinogenic effects and cause respiratory, cardiovascular, and neuropsychiatric adverse events (*1*). In 2019, a form of ketamine (esketamine) was approved by FDA for use in treatment-resistant depression among adults^†^ (*2*). Ketamine use, poison center calls for ketamine exposure, and ketamine drug reports from law enforcement have increased through 2019 (*3*), but recent trends in ketamine involvement in fatal overdoses are unknown. Data from CDC’s State Unintentional Drug Overdose Reporting System (SUDORS) were analyzed to describe characteristics of and trends in overdose deaths with ketamine detected or involved during July 2019–June 2023.

## Investigation and Findings

Data on drug overdose deaths with unintentional or undetermined intent come from SUDORS, which includes information from death certificates, medical examiner or coroner reports, and postmortem toxicology reports.^§^ Data are abstracted on all substances reported to cause death (i.e., involved) and substances detected through toxicology testing.^¶^ Decedent demographics and other overdose characteristics were analyzed among 45 jurisdictions (44 states and the District of Columbia [DC]),** and trend analyses were conducted among 28 jurisdictions (27 states and DC).^††^ Analyses were restricted to deaths with toxicology reports or with ketamine listed as a cause of death on the death certificate. Ketamine detection included toxicology results for ketamine or its metabolites.^§§^ Among deaths with ketamine detected, drug involvement was analyzed to ascertain which drug or drugs caused death. This activity was reviewed by CDC, deemed not research, and was conducted consistent with applicable federal law and CDC policy.^¶¶^

During July 2019–June 2023, a total of 228,668 drug overdose deaths were identified in 45 jurisdictions. Ketamine was detected in 912 (0.4%) overdose deaths, listed as involved in 440 (0.2%) deaths, and was the only substance involved in 24 (0.01%) deaths (Table). A majority of deaths with ketamine detected involved illegally manufactured fentanyls (IMFs) (58.7%), followed by methamphetamine (28.8%) and cocaine (27.2%). Overall, 82.4% of deaths involved either IMFs, methamphetamine, or cocaine. Approximately one third (34.8%) of decedents in whom ketamine was detected were aged 25–34 years, and approximately three quarters were males (71.3%) and non-Hispanic White persons (73.7%).

**TABLE:** Characteristics of drug overdose deaths with ketamine* detected**^†^** (N = 912) — State Unintentional Drug Overdose Reporting System,**^§^** 45 U.S. jurisdictions, July 2019**–**June 2023

Characteristic	No. (%) of ketamine-detected deaths
**Age group, yrs**	
≤14	3 (0.3)
15–24	117 (12.8)
25–34	317 (34.8)
35–44	223 (24.5)
45–54	125 (13.7)
55–64	102 (11.2)
≥65	25 (2.7)
**Sex**	
Female	262 (28.7)
Male	650 (71.3)
**Race and ethnicity** ^¶^	
American Indian or Alaska Native, non-Hispanic	21 (2.3)
Asian, non-Hispanic	24 (2.6)
Black or African American, non-Hispanic	97 (10.7)
Native Hawaiian or Pacific Islander, non-Hispanic	0 (—)
White, non-Hispanic	671 (73.7)
Hispanic or Latino	73 (8.0)
Multiple races, non-Hispanic	19 (2.1)
Drugs involved in overdose**	
Ketamine listed as cause of death (ketamine-involved)	440 (48.2)
No other drugs involved (ketamine only)	24 (2.6)
IMFs,^††^ methamphetamine, or cocaine	751 (82.4)
IMFs^††^	535 (58.7)
Methamphetamine	263 (28.8)
Cocaine	248 (27.2)
Benzodiazepines	162 (17.8)
Prescription opioids	129 (14.1)
Alcohol	121 (13.3)
Antidepressants	45 (4.9)

**Abbreviation:** IMFs = illegally manufactured fentanyls.

* Ketamine was considered to be detected if one or more of the following parent drugs or metabolites were identified by postmortem toxicology testing: ketamine, dehydronorketamine, hydroxynorketamine, norketamine, and ketamine metabolite (not otherwise specified).

^†^ A drug was considered to be detected if it was present in the postmortem toxicology report. A detected drug might or might not be listed as a cause of death (i.e., involved).

^§^ The following 45 jurisdictions had complete toxicology results for ≥75% of deaths for any 6-month reporting period during July 2019–June 2023: Alabama, Alaska, Arizona, Arkansas, Colorado, Connecticut, Delaware, District of Columbia, Florida, Georgia, Hawaii, Illinois, Indiana, Iowa, Kansas, Kentucky, Louisiana, Maine, Maryland, Massachusetts, Michigan, Minnesota, Mississippi, Missouri, Montana, Nebraska, Nevada, New Hampshire, New Jersey, New Mexico, New York, North Carolina, Ohio, Oklahoma, Oregon, Pennsylvania, Rhode Island, South Dakota, Tennessee, Utah, Vermont, Virginia, Washington, West Virginia, and Wisconsin.

^¶^ Missing values were excluded from calculations of percentages. Percentages might not sum to 100% because of rounding.

** A drug overdose can involve multiple drugs. Consequently, specific drug percentages when summed will exceed 100%.

^††^ IMFs were classified as likely illegally manufactured using toxicology, scene, and witness evidence. In the absence of sufficient evidence to classify fentanyl as illegal or prescription, fentanyl was classified as illegally manufactured because the majority of fentanyl overdose deaths involve IMFs. All fentanyl analogs except alfentanil, remifentanil, and sufentanil (which have legitimate human medical use) were included as IMFs.

Among 172,475 overdose deaths in 28 jurisdictions during July 2019–June 2023, <1% had ketamine detected (692 deaths; 0.4%) or were classified as ketamine-involved (348 deaths; 0.2%). The number and percentage of deaths with ketamine detected increased during July 2019–June 2023 from 47 (0.3%) to 107 (0.5%), with notable increases as early as July–December 2020 (Supplementary Figure, https://stacks.cdc.gov/view/cdc/168876).

## Conclusions and Actions

During July 2019–June 2023, although ketamine was detected or involved in <1% of all drug overdose deaths, overdose deaths with ketamine detected increased. Almost all overdose deaths with ketamine detected involved other substances, mostly IMFs or stimulants; however, the source of ketamine (e.g., illegally purchased or prescribed) is unknown. Because analyses included a subset of jurisdictions, findings might not be generalizable to the entire United States. In addition, the scope of postmortem toxicology testing varies within and across jurisdictions, and ketamine might not be included in testing panels or be tested for in all postmortem samples (*4*), which could lead to an underestimation of ketamine detection. Despite the lack of uniform testing, ketamine detection among overdose deaths has increased over time, yet both detection and involvement accounted for a small proportion of overdose deaths. As polysubstance use (*5*) and use of ketamine for treatment-resistant depression and in compounded formulations*** increase, continued monitoring is needed to identify potential changes in the detection and involvement of ketamine in overdose deaths and to better understand potential drug interactions or circumstances leading to death.

## Footnotes

* Drugs, substances, and certain chemicals used to make drugs are classified into five distinct categories or schedules depending upon the drug’s acceptable medical use and the drug’s abuse or dependency potential. Schedule III substances are defined as those that have moderate to low potential for physical and psychological dependance. https://www.dea.gov/drug-information/drug-scheduling

†
https://www.fda.gov/news-events/press-announcements/fda-approves-new-nasal-spray-medication-treatment-resistant-depression-available-only-certified

§
https://www.cdc.gov/overdose-prevention/data-research/facts-stats/about-sudors.html

¶ A drug was considered involved if it was listed as a cause of death on the death certificate or medical examiner or coroner report. A drug was considered detected if it was present on the postmortem toxicology report. A detected drug might or might not be listed as a cause of death.

** The following 45 jurisdictions had complete toxicology results for ≥75% of deaths for any 6-month reporting period during July 2019–June 2023: Alabama, Alaska, Arizona, Arkansas, Colorado, Connecticut, Delaware, District of Columbia, Florida, Georgia, Hawaii, Illinois, Indiana, Iowa, Kansas, Kentucky, Louisiana, Maine, Maryland, Massachusetts, Michigan, Minnesota, Mississippi, Missouri, Montana, Nebraska, Nevada, New Hampshire, New Jersey, New Mexico, New York, North Carolina, Ohio, Oklahoma, Oregon, Pennsylvania, Rhode Island, South Dakota, Tennessee, Utah, Vermont, Virginia, Washington, West Virginia, and Wisconsin.

†† The following 28 jurisdictions had complete death certificate and toxicology results for ≥75% of deaths for all 6-month reporting periods during July 2019–June 2023, allowing for trend analyses: Alaska, Arizona, Colorado, Connecticut, Delaware, District of Columbia, Georgia, Illinois, Kansas, Kentucky, Louisiana, Maine, Maryland, Massachusetts, Minnesota, Nebraska, Nevada, New Hampshire, New Jersey, Ohio, Oklahoma, Oregon, Pennsylvania, Rhode Island, Utah, Vermont, Virginia, and Washington.

§§ Ketamine overdose deaths included mention of the following parent drug, metabolites, or analogs in toxicology reports: ketamine, dehydronorketamine, hydroxynorketamine, norketamine, and ketamine metabolite (not otherwise specified).

¶¶ 45 C.F.R. part 46.102(l)(2), 21 C.F.R. part 56; 42 U.S.C. Sect. 241(d); 5 U.S.C. Sect. 552a; 44 U.S.C. Sect. 3501 et seq.

*** https://www.fda.gov/drugs/human-drug-compounding/fda-alerts-health-care-professionals-potential-risks-associated-compounded-ketamine-nasal-spray; https://www.fda.gov/drugs/human-drug-compounding/fda-warns-patients-and-health-care-providers-about-potential-risks-associated-compounded-ketamine
